# To Kill or to Be Killed: How Does the Battle between the UPS and Autophagy Maintain the Intracellular Homeostasis in Eukaryotes?

**DOI:** 10.3390/ijms24032221

**Published:** 2023-01-22

**Authors:** Peifeng Yu, Zhihua Hua

**Affiliations:** 1 Department of Environmental and Plant Biology, Ohio University, Athens, OH 45701, USA; 2 Interdisciplinary Program in Molecular and Cellular Biology, Ohio University, Athens, OH 45701, USA

**Keywords:** ubiquitin, proteasome, autophagy, ubiquitylation, protein degradation, liquid–liquid phase separation, biophysical state, development, stress

## Abstract

The ubiquitin-26S proteasome system and autophagy are two major protein degradation machineries encoded in all eukaryotic organisms. While the UPS is responsible for the turnover of short-lived and/or soluble misfolded proteins under normal growth conditions, the autophagy-lysosomal/vacuolar protein degradation machinery is activated under stress conditions to remove long-lived proteins in the forms of aggregates, either soluble or insoluble, in the cytoplasm and damaged organelles. Recent discoveries suggested an integrative function of these two seemly independent systems for maintaining the proteome homeostasis. One such integration is represented by their reciprocal degradation, in which the small 76-amino acid peptide, ubiquitin, plays an important role as the central signaling hub. In this review, we summarized the current knowledge about the activity control of proteasome and autophagosome at their structural organization, biophysical states, and turnover levels from yeast and mammals to plants. Through comprehensive literature studies, we presented puzzling questions that are awaiting to be solved and proposed exciting new research directions that may shed light on the molecular mechanisms underlying the biological function of protein degradation.

## 1. Introduction

Protein misfolding is a harmful post-translational process that could permanently damage the activity of individual proteins and protein complexes, thus reducing cellular fitness [1]. Many aberrant intracellular processes, including genetic mutations, incomplete translation, abnormal folding after translation, mis-regulated post-translational modifications, oxidative damage, and defective assembly of protein complexes, can lead an intracellular peptide to fold abnormally [2]. In many cases, protein misfolding exposes the main chain and the hydrophobic patches of a protein that are largely buried internally within the normally folded structure. The exposure of these regions triggers aggregation, but can also sequester normally folded proteins, thus perturbing the proteome function [3]. An unhealthy proteome has catastrophic consequences at the organismal level, such as causing numerous human proteinopathies [4,5], and resulting in stress and even developmental termination in plants [6]. 

Since protein misfolding is an inevitable and generic feature of polypeptides due to a crowded and busy intracellular environment [3], eukaryotic cells have developed multilayers of protein quality control (PQC) machineries to defend protein misfolding [7]. The forefront layer relies on chaperone-assisted refolding or the direct turnover of abnormally folded proteins through the ubiquitin-26S proteasome system (UPS) to prevent them from aggregation. However, aggregation can still occur if misfolded proteins are overwhelmed and beyond to be repaired or degraded individually. At this point, an autophagy-mediated lysosomal/vacuolar degradation system would be activated to serve as the last defense system to remove potentially toxic protein aggregates [2].

Since the seminal work of Finley et al. in 1984 on the discovery of ubiquitin-dependent protein turnover and the later discovery of proteasomal degradation of ubiquitylated proteins by Heinemeyer et al. in 1991 [8,9], the UPS-mediated protein degradation has been widely appreciated as the major protein degradation machinery in all eukaryotic organisms. It was estimated that as much as 80–90% of protein turnover in cultured mammalian cells was accounted for by UPS-mediated protein degradation under optimal nutritional conditions [10], further suggesting the strong evolutionary selection against protein aggregation [3]. As an alternative degradative system, inhibitor studies have also demonstrated the contribution of an autophagy-mediated lysosomal/vacuolar degradation system in 10–20% of long-lived protein degradation upon nutrient (serum) deprivation [11]. 

Throughout the past 40 years of research, overwhelming studies have concluded the tight connection between the activities of the UPS and autophagy and numerous human protein aggregation diseases. Not only are a vast number of abnormal and short-lived proteins, including 30% of newly synthesized cellular proteins, targeted by the UPS, but also multitype autophagy substrates, such as protein aggregates and defective organelles, have been identified for lysosomal/vacuolar degradation. It was also found that the two systems coordinate and interplay in the surveillance of PQC. For example, the inhibition of one system may upregulate the other or vice versa. For detailed mechanisms of each system, the readers are referred to excellent review articles in the field [12,13,14,15,16,17,18,19,20,21,22,23,24]. Because of space limitation, in this review, we focus on the current knowledge about how the two systems are regulated through their structural organization and reciprocal degradation. Whenever possible, we referred to the studies in plant biology research, with some knowledge gaps filled with the discoveries in other systems, such as yeast and mammalian cell lines.

## 2. Quality Control of the 26S Proteasome

### 2.1. Structure and Activity Control of the 26S Proteasome

**Figure 1:**
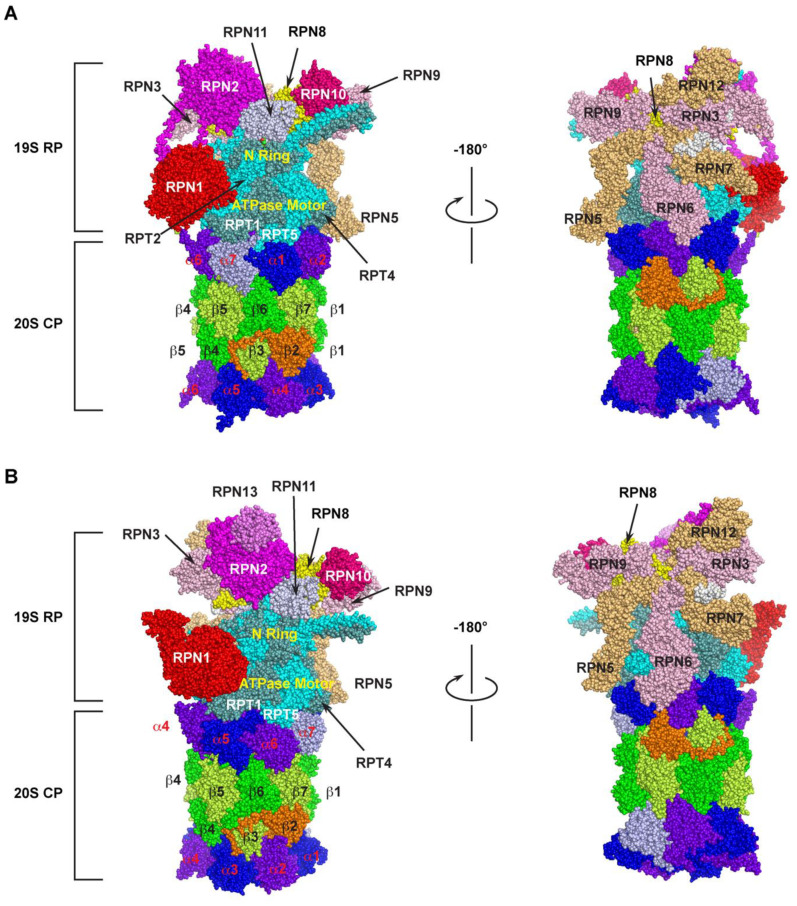
Cryo-electron microscopy (Cryo-EM) structure of the 26S proteasome. The spherical structure is generated based on PDB 6MSK [36] and 3JCP [37] for humans (**A**) and yeast (**B**) 26S proteasome, respectively. Two mirror images were shown for the detailed organization of base and lid subunits of the 19S RP subcomplex in each proteasome. The parts of N-ring and ATPase motor ring of RPT hexameric complex are indicated. The subunits of α-ring and β-ring of the 20S CP are highlighted with different colors. The human 26S proteasome structure does not include RPN13.

The 26S proteasome is one of the largest multi-subunit protein complexes that is composed of two functionally distinguished subcomplexes (Figure 1). The proteolytic part is attributed to a barrel-shaped 20S core particle (CP) that comprises four axially stacked heteroheptameric rings (two outer α- and two inner β-rings) [25]. Among the four rings, three of the seven pairs of the β subunits (β1, β2, and β5) possess six catalytic sites that have caspase-like (cleave after acidic amino acid), trypsin-like (post-basic), and chymotrypsin-like (post-hydrophobic) specificities, giving the proteolytic function of the 26S proteasome [26]. These subunits utilize the hydroxyl group of the terminal threonine residue as the catalytic nucleophile for attacking peptide bonds of a substrate [10]. However, the 20S CP alone does not have the proteolytic function, because the N-termini of the seven pairs of the α-ring subunits completely blocks the accessibility of unregulated substrates to the proteolytic chamber by sealing the entrance pore (“gate”) on both sides of the internal β-rings [27,28,29,30]. 

The 19S regulatory particle (RP) is designed to recognize the substrate, remove ubiquitin modifications, control the opening of the substrate entrance pore, unfold, and translocate the substrate into the 20S core. It binds to either or both ends of the 20S CP to assemble a 26S or 30S proteasome holo-complex, respectively, based on their sedimentation coefficients [31,32]. According to organization and function, a typical RP can be further divided into a base and a lid subcomplex [33,34,35].

The base is made up of 10 subunits, including four Regulatory Particle Non-ATPase (RPN)1 [38], RPN2 [39,40], RPN10 [41], and RPN13 [42], and a hexameric ATPase motor ring composed of six Regulatory Particle Triple-A ATPases (RPT) 1–6 [43,44] (Figure 1). In addition to the ATP hydrolysis function of the Triple-A ATPases active domain, the RPT subunits carry an N-terminal oligonucleotide/oligosaccharide-binding (OB)-fold domain [45,46,47] and a C-terminal HbYX motif (where Hb stands for a hydrophobic residue; Y for tyrosine; and X for any amino acid) [48]. The OB domains form a rigid N-terminal ring that is stacked on top of the AAA-domain ring [38,44,46]. Upon recognition, a ubiquitylation substrate needs to go through the N-ring before engaging with the AAA-ATPases that convert ATP hydrolysis energy into mechanical pulling for its unfolding and translocation into the 20S core [47,49,50]. It was shown that an unstructured initiation region with at least 20–25 amino acids is required for a substrate to be engaged with the ATPase motor [47,51,52,53,54,55,56,57]. This necessity may provide the second degradation code for a ubiquitylation substrate in addition to the topology of polyubiquitin chains. The C-terminal HbYX motif of an RPT subunit binds to a pocket between the 20S CPs α subunits functioning as a ‘‘key in a lock’’ to induce gate opening and allow substrate entry [48,58,59]. Two of the four RPN subunits, RPN10 and RPN13, function as the receptors of ubiquitylation substrates [42,43]. RPN1 and RPN2 are the two paralogues with the largest size among the entire group of the 26S subunits [60,61]. Their central α-turn-α proteasome/cyclosome (PC) motifs form a toroid structure that is further extended by their divergent flexible N- and C-terminal regions. The large size and the toroid structure give them a role in functioning as flexible scaffolds between the base and the lid [40,62,63,64] (Figure 1A). The different positions of RPN1 and RPN2 within the 19S RP also fulfil their specific functions. For example, RPN1 also functions as the third receptor for the poly-ubiquitin chain to facilitate deubiquitylation [38,43,60], while RPN2 tightly interacts with RPN13 and a proteasome-associated DUB, UCH37/UCH-L5 [65,66,67] (Figure 1B). RPN2 was also shown to bind with importin αβ for mediating nuclear imports of proteasomal components [68].

The RP lid contains nine subunits that include RPN3, RPN5-9, RPN11-12, and RPN15 [69] (Figure 1). Among them, RPN11 is a Zn^2+^-dependent deubiquitylase (DUB) [44,70,71], forming a hetero-dimeric complex with RPN8 [72,73]. The six Proteasome–CSN–eIF3 (PCI) domains containing subunits, RPN3, 5, 6, 7, 9, and 12, assemble a horseshoe structure playing a scaffolding function to facilitate inter-subunit binding [34,35,61] (Figure 1A,B, right panel). RPN8 sits in the horseshoe through interacting with RPN3 and RPN9. It also forms a heterodimer with RPN11, projecting the active site of RPN11 near the N-ring of RPTs (Figure 1A-B, left panel). The C-terminus of RPN3 was shown to contact the N-ring of the RPT base proposed to form a composite active site for substrate deubiquitylation and unfolding [61,73]. Thus, the C-terminus of RPN3 may function as a sensor of substrates engaged in the N-ring for initiating conformational changes of the lid to activate RPN11 and the composite active site [61,74].

### 2.2. Composition of the Plant 26S Proteasome

The past decade of structural studies have shed rich insights into the dynamic function of substrate recognition, engagement, and degradation in a functional proteasome of yeast [34,47,74,75,76,77,78,79], humans [36], and mammals [80]. Unfortunately, no structural information has been available for a plant proteasome, although it has been clearly demonstrated that the UPS plays a tremendous importance in plant evolution, growth, and development [15,17,81,82,83,84]. The lack of such a critical study in understanding the kinetics and structure of the major degradation machinery in plants is reminiscent of a continuing issue of the weak attention on recognizing plant science as an important discipline for discoveries in basic biology [85]. Nevertheless, the pioneering work conducted in the Vierstra lab has uncovered multiple conserved and specific mechanisms of plant proteasomes, including the first discovery of proteaphagy (see below).

Through early genomic studies, 23 genes encoding a complete list of 14 20S CP proteasome subunits [86], 11 genes for RPT1-6 [87], and 6 genes for non-ATPase subunits (RPN1, 2, 6, 8, 10, and 11) [88], were identified in Arabidopsis, primarily in the representative Columbia-0 accession. In addition to gene identification, genomic DNA analysis by Southern blotting confirmed gene duplications for multiple CP and RP genes [86,87]. The biochemical function of some gene products, including three CP genes, *PAC1* (α3), *PAE1*(α5), and *PBC2* (β3) [86], and five *RPT* genes, *RPT1* and *3–6*, were confirmed by complementation assay in yeast that lacked the expression of a corresponding orthologous gene. However, the big picture about the Arabidopsis holo-proteasome complex was lacking by this assay, due to an incomplete genome sequence database and the unavailability of comprehensive evolutionary studies. 

**Figure 2:**
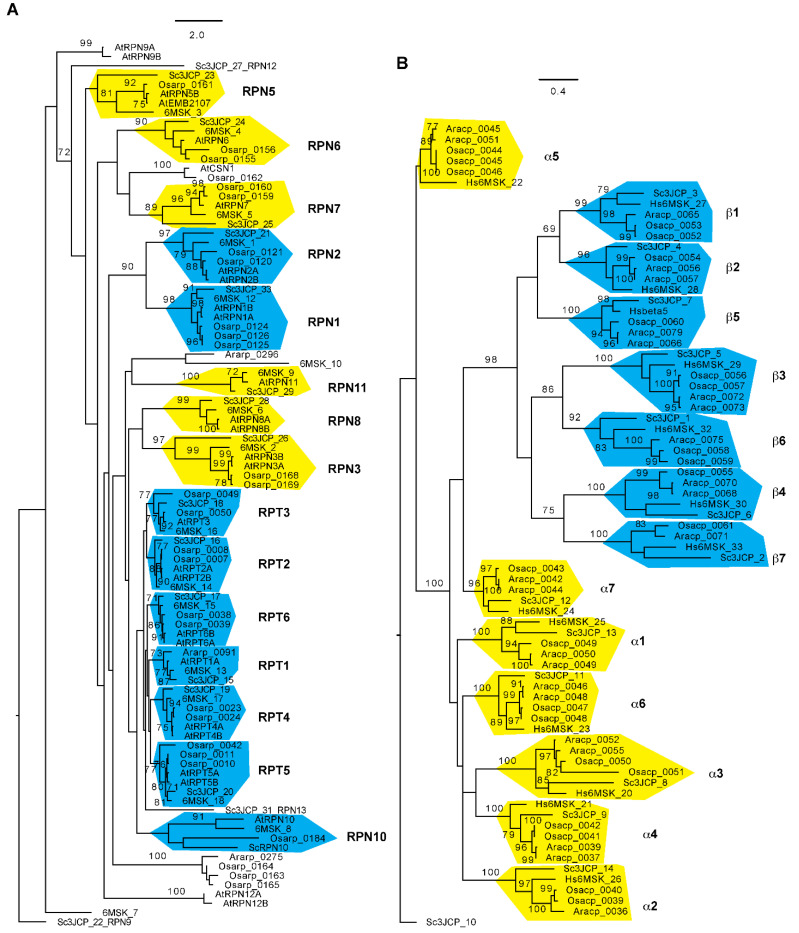
Phylogenetic relationship of 26S proteasome subunits among Arabidopsis, rice, yeast, and humans. The sequences and sequence identifications of Arabidopsis and rice 26S proteasome subunits were retrieved from Hua and Yu, 2019 [89], and those for yeast and human 26S proteasomes were obtained from Luan et al. (2016) [37] and Dong et al. (2019) [36], respectively. The RP (**A**) and CP (**B**) phylogenetic trees were separately generated using RAxML (Version 8.1) with the PROTGAMMAJTT substitution model [97]. A significant clade is indicated with a bootstrap value greater than 70% that was calculated based on 1000 replications. Independent clades are labeled and shaded with yellow ((**A**): lid subunits; (**B**): α subunits) or cyan ((**A**): base subunits; (**B**): β subunits) color. Scale bar: average substitutions per site. Ara: Arabidopsis; Osa: *Oryza sativa*; Hs: *Homo sapiens*; Sc: *Saccharomyces cerevisiae*.

Using an improved bioinformatic analysis pipeline, we were able to find genes de novo for 30 out of all 34 proteasome subunits in seven flowering plants that include two evolutionary distant genera, Oryza and Arabidopsis, each with three species, and the basal flowering plant, *Amborella trichopoda* [89]. RPN11, 12, 13, and 15 were not studied because the simplicity of their domain structures was not helpful for distinguishing them from members of the eIF3 and CSN complexes. Our genomic and evolutionary studies uncovered a genus/species-specific feature of plant proteasomes. For example, no orthologues of *RPN8* were identified in three Oryza species. Since the *Oryza sativa* Nipponbare reference genome represents the second best-annotated plant genome [90], the failure in finding an *RPN8* orthologue in the rice genome is not likely due to genome sequence errors. We also discovered that most proteasome members from the two genera form an independent subclade with strong statistical significance support, further suggesting the functional diversification of plant proteasomes [89] (Figure 2). How this evolutionary divergence contributes to the biochemical and functional specialization of proteasomes requires further investigation.

It was a breakthrough discovery in proteosome biochemistry studies to functionally replace a proteosome subunit with a tagged recombinant version [91]. This replacement not only allows for the affinity purification of proteome complexes in vivo for proteomics studies, but also develops proteasomal reporters for monitoring intracellular dynamics of proteasomes through live cell imaging. For example, the Vierstra lab has developed three different Arabidopsis transgenic lines, in which the endogenous CP subunit PAG1 (α7) and two isoforms of the RP subunit RPT4 were replaced with FLAG-tagged fusions. Using a C-terminal tagged PAG1-FLAG in conjugation with in-depth mass spectrometry proteomic sequencing, the two duplicated isoforms of most 26S subunits were identified, suggesting the presence of a heterogeneous population of 26S proteasome particles in Arabidopsis [92]. This raised an intriguing question as to whether the plant proteasomes are assembled in an isoform-specific manner. To address this question, the group cleverly designed two N-terminal FLAG-tagged RPT4 isoforms to develop *FALG-RPT4a rpt4a-1* and *FALG-RPT4b rpt4b-2* transgenic Arabidopsis plants. Utilizing these two transgenic plants and a similar affinity purification-based mass spectrometry proteomics, the authors failed to identify 26S proteasome isotypes that are specific to the RPT4 paralogues. This seems to be contradictory to the case of mammalian proteasomes, in which different paralogues of β1, β2, and β5 subunits were found in specialized proteasome isoforms with varied proteolytic activities, such as immunoproteasomes and thymoproteasome [93,94]. Since these specialized mammalian proteasomes are present in a tissue, organ, and physiology-specific manner [93], the failure of discovering paralogue-specific 26S proteasome isotypes in Arabidopsis seedlings does not mean that there is an absence of such types of proteasomes in plants. Indeed, we recently discovered that the proteasomes in seedlings and developing siliques possess different catalytic activities and sensitivities to the inhibition of MG132, strongly indicating their presence in plants [95]. Nevertheless, such an affinity for purification-based proteomic studies have discovered the complete set of Arabidopsis proteasome subunits that are orthologous to the yeast and mammalian counter partners as well as a rich group of proteasome-associated proteins, including proteasome assembly chaperons, a PA200/Blm10 regulator that caps the CP, and a ubiquitin-binding shuttle factor DSK2 [92,96].

### 2.3. Post-Assembly Regulation of Proteasomes

Due to the space limit, this review does not focus on the dynamic synthesis of a 26S proteasome complex. For this topic, the readers are referred to recent review articles written by Marshall and Vierstra [98], Yedidi et al. [99], Enenkel [31], Mao [100], Budenholzer et al. [101], and Kato and Satoh [102]. Given the large composition, it is energetically costive to assemble and maintain the equal stoichiometry of a minimum of 34 proteasome subunits in a holo-complex (Figure 1 and Figure 2). The requirements of a high-energy investment can inevitably result in errors in both assembling the complete complex and maintaining the functionality of the holo-complex when unfavorable conditions occur. Such stress conditions include, but are not limited to, nutrient starvations, oxidative damages, inhibitory effects, defective subunits resulting either from genetic mutations or translational errors, and subunit misassembly [103]. In this section, we discuss how post-assembled proteasomes with different biophysical states are regulated to meet the requirements of cellular growth and development. 

#### 2.3.1. Proteasome Storage Granules (PSGs)

Through labelling a proteosome subunit with a fluorescent marker, such as a green fluorescent protein (GFP), it is possible to monitor the dynamic localization of a proteasome. For example, most yeast proteasomal subunits can be chromosomally replaced with a GFP-tagged variant. Using this strategy, it was shown that ~80% of the 26S proteasomes were nucleus-localized in actively dividing yeast and mammalian cells [31,104,105]. The nuclear presence of the GFP-labeled proteasome maturation factor Ump1 suggests that some particles represent 20S CP precursor complexes that are in the process of maturation in the nucleus. Thus, the major proportion of both mature and immature proteasomes are present in the nucleus.

Cells are growing under a dynamic shifting environment, which are in many cases suboptimal or even unfavorable. These stress environmental signals can trigger different responsive programs to reprogram intracellular metabolism. The proteasome activities are unequivocally regulated by these signals. Upon carbon starvation, yeast cells enter quiescence [106]. Along with a large fraction of metabolic proteins forming reversible macroscopic foci in quiescent cells [107], nucleus-localized proteasomes were also discovered to dissociate into CP and RP particles to be exported into the cytoplasm to form so-called proteasome storage granules (PSGs) [108]. It was shown that both CP and RP are required to form PSGs [109,110]. However, no holo-complexes are assembled due to declined ATP levels [111]. Thus, PSGs are inactive. In yeast, CP is found to be associated with Blm10, which may also prevent its reassembly with RP [112]. 

Through high-throughput microscopy on a collection of yeast null-mutants in combination with proteomic studies, the yeast PSGs were discovered to contain not only disassembled CP and RP particles, but also a significant proportion of free ubiquitin molecules that are essential for the nuclear export and sequestration of proteasomes into PSGs [113]. Because all components in the PSGs can revert their biological functions upon the onset of cell proliferation or growth, the PSGs are considered as reservoirs for both proteasome and ubiquitin, for cells to survive under an austerity budget of energy [113]. PSGs represent one type of protein droplets formed through a process now widely recognized as liquid–liquid phase separation (LLPS) [114]. However, because LLPS often requires the presence of intrinsically disordered protein–protein interaction domains that are rare in proteasome subunits [115], the detailed mechanism in the formation of PSGs remain an enigma [116]. It is also unclear whether plants utilize PSGs as a defensive mechanism to overcome carbon starvation. Plant proteasomes seem to be equally localized in both the nucleus and cytoplasm for actively growing seedlings [117,118]. Whether and how the CP and RP subcomplexes in the nucleus and cytoplasm are dissembled simultaneously and intermingled into the cytoplasmic PSGs remains largely unknown.

#### 2.3.2. Proteaphagy

Unlike carbon starvation, nitrogen starvation triggers the autophagy-mediated vacuolar and lysosomal degradation of proteasomes in plants/yeast and mammalian cells, respectively [118,119,120]. It was also found in plants that the treatment of a tri-peptide aldehyde proteasome inhibitor, carbobenzoxy-Leu-Leu-leucinal (MG132), induced the degradation of the holo-proteasome complex that requires the intact autophagic activity [118]. The discovery of nutrient starvation, genetic aberration, and chemical inhibition-induced autophagic degradation of whole proteasomes in plants led to the term “proteaphagy”, first coined by Marshall et al. [118]. Further studies from the group found that nitrogen depletion-induced proteaphagy differed from that induced by MG132 inhibition. While the authors speculated that nitrogen depletion-induced proteaphagy is regulated through a general bulk autophagy pathway, they uncovered RPN10 as a new selective receptor that specifically bridges the MG132-inhibited proteasomes into autophagosome through interaction with both heavily ubiquitylated proteasomes and AuTophaGy (ATG)8 [118]. The discovery of proteaphagy provides new evidence showing the integration of the two major protein degradation pathways in PQC [1]. 

However, many unaddressed questions remain. For example, the nitrogen depletion-induced plant proteaphagy is independent on known autophagy cargo receptors, including next to BRCA1 (NBR1) and RPN10, which is suggestive of a general autophagy process [118]. However, P62, a receptor binding both polyubiquitin chains and microtubule-associated protein 1 light chain 3 (LC3; the mammalian ATG8 orthologue) proteins, is required for amino acid starvation-induced proteaphagy in mammalian cells [119]. Through both pharmacological treatment and nitrogen starvation assays, proteaphagy in yeast was also discovered to be selective and it required factors not involved in general autophagy [120]. Plant proteaphagy can be hijacked by bacteria for enhancing virulence through degrading proteasomes. This type of proteaphagy was shown to be induced by a type III effector, HopM1, from bacterial cells, and was activated in a manner comparable to MG132 inhibition. However, whether RPN10 functions as a receptor is yet unclear. Interestingly, NBR1-dependent selective autophagy, albeit activated by bacterial infection, counteracts disease progression. The authors suggested that NBR1 contributes to the turnover of ubiquitylated substrates that were hyperaccumulated during bacterial infection [121]. Plant proteaphagy can also be activated developmentally. Through monitoring the daily changes of proteasome subunits, ubiquitylated proteins and autophagy activities in developing siliques upon pollination for an 8-day developmental period, we recently discovered a relay model of the two degradation pathways in regulating silique/seed development in Arabidopsis. We uncovered that proteaphagy is activated when late heart embryos are developed. Given the same developmental trend of silique proteaphagy in wild types and the *rpn10-1* mutant (expressing a truncated RPN10 unable to bind ubiquitylated substrates), the silique proteaphagy is not likely to be selected by RPN10 either. Whether this is due to a general bulk autophagy degradation or NBR1-dependent selective autophagy requires further investigation [95].

The detailed upstream signaling of proteaphagy is not clear either. For example, why do nitrogen depletion and carbon starvation result in different outcomes of proteasome regulation, although both activate autophagy pathways through the master nutrient sensor TORC1? The former induces proteasome degradation through proteaphagy [118,119,122], while the latter prevents the elimination of proteasomes through PSGs [108]. Interestingly, proteaphagy upon nitrogen starvation in yeast involves CP and RP dissociation, nuclear export, and the independent vacuolar targeting of CP and RP subcomplexes. The inhibition of proteaphagy leaves most RPs in the nucleus but discharges CPs into cytosol either as free forms or to be localized into the granular structures within the cytosol [122]. Since the formation of PSGs requires both CP and RP complexes [109,110], can these granular structures be PSGs formed upon carbon starvation? It is yet unknown whether nitrogen starvation, like amino acid starvation in mammalian cells, enhances the ubiquitylation of proteasomes in yeast and plants. Affinity-purified proteasomes in seedlings treated with or without nitrogen starvation did not result in any significant changes of proteasome ubiquitylation [118]. Does this difference result from sequence divergence of proteasome subunits across different species and kingdoms? 

#### 2.3.3. Aggrephagy

Misfolded or malfunctioning proteins may coalesce into perinuclear and MTOC-localized aggresomes through dynein-dependent retrograde transport on microtubes in mammalian cells [7]. In addition, they have also been found in both yeast and mammalian cells to form two other MTOC-independent aggresome-like structures, a soluble juxtanuclear quality control (JUNQ) compartment and an insoluble protein deposit (IPOD) compartment [123]. Since misfolded proteins are cytotoxic, sequestering them into proteinaceous compartments not only provides cytoprotective functions, but also accelerates their clearance by activating UPS and/or autophagy-lysosome/vacuole degradation machineries [2,7,123]. Clearance of cytosolic aggregated by autophagy-lysosomal/vacuolar degradation is generally termed as aggrephagy [2].

Impaired proteasomes by either MG132 reverse inhibition or partially genetic mutations have been found in mammalian aggresomes and yeast IPOD droplets, respectively. Both inclusions were targeted by autophagy-mediated lysosomal or vacuolar degradations [124,125]. The formation of proteasome-containing aggresomes and IPOD droplets was verified by the requirements of HDAC6- and dynein-mediated transport and the colocalization with IPOD markers (Hsp104 and Rnq1), respectively [124,125]. Although JUNQ and IPOD were initially defined by the presence and absence of ubiquitylated proteins, respectively [123], ubiquitylated proteasomes were found to be sequestered into IPODs when the proteasome functions were completely blocked by genetic aberration in yeast [125]. 

**Figure 3:**
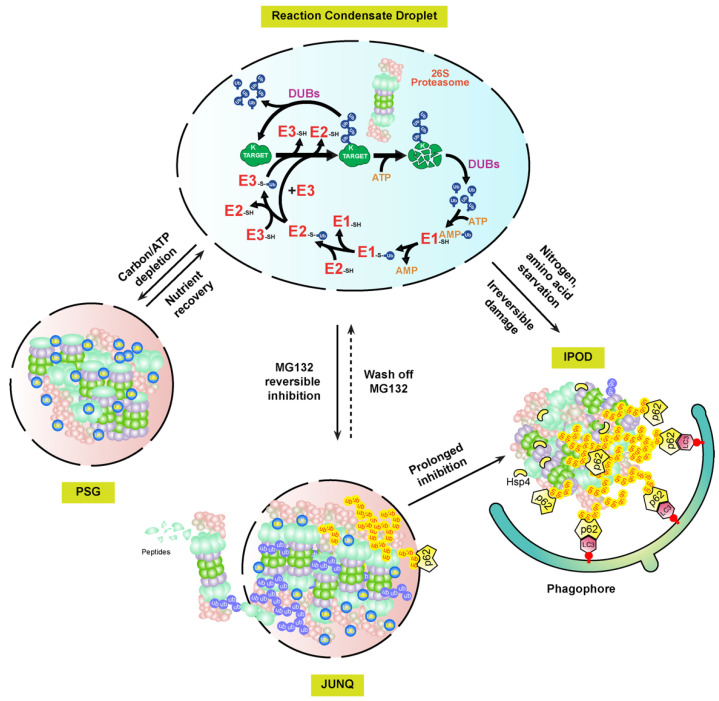
A biophysical dynamic module of intracellular proteasomes with different functional activities and stabilities. In normal cellular growth conditions, the UPS and its substrates may develop concentrated liquid droplets through LLPS for efficient proteolysis of ubiquitylation substrates [127]. Upon different stresses, the soluble proteasomes may either form PSG or JUNQ condensates, depending on whether the RP and CP subcomplexes are dissociated in yet unknown mechanisms [108,124]. The substances, including damaged proteasomes, in the highly fluidic JUNQ body are still under active ubiquitylation reactions. Ubiquitylated proteasome subunits may be extracted out of the JUNQ body for degradation by a functioning 26S proteasome, or directly targeted for autophagy-lysosomal/vacuolar degradation in either piecemeal or wholesale versions via autophagy receptors, e.g., p62 [128]. Continuing structure disruption by harsh or prolonged stresses may terminally sequester proteasome aggregates into IPOD foci, which are mediated by Hsp4 and only recognized by autophagy for selective degradation [125]. Proteasomes targeted by proteaphagy could be from either JUNQ or IPOD aggregates.

Interestingly, the proteasome-containing aggresomes formed by reversible MG132 inhibition in mammalian cells are under both autophagy clearance and proteasome reversion processes, suggesting that they are JUNQ-like fluidic foci, but not IPODs [124]. Given that PSGs function as cytoplasmic storage foci for normal proteasomes that can be fully reverted when carbon starvation is removed [108], these MG132-induced proteasome fluidic aggresomes may function as a temporary storage place for malfunctioning proteasomes awaiting recovery (Figure 3). The requirement of proteasomal ubiquitylation by STUB1 in the aggresomal formation of inhibited proteasomes may play a similar role of the free ubiquitin in PSGs, which are not only essential for the formation but also for keeping the fluidity. Given the presence of DUB activities by RPN11 and other DUB enzymes associated with proteasomes, a high concentration of free ubiquitin could be enriched in these foci. However, prolonged or more severed stresses, such as proteasome inhibition by irreversible inhibitors, epoxomicin and carfilzomib, or genetic aberrations, would completely block the activity of the holo-complex, thus changing the fluidity of the aggresome for converting into IPODs.

The presence of two fluidity statuses of mammalian proteasome-containing aggresomes, JUNQ and IPOD, implies that plant proteasome aggregates may have similar versions. For example, early plant growth inhibition by MG132 can be rapidly recovered upon being transferred to normal growth media. This may reflect the presence of JUNQ-like proteasome aggregates that are targeted by RPN10-medaited proteaphagy in MG132-treated seedlings [118] (Figure 3). Up to now, we still do not know how plant proteasome aggregates are formed. Clearly, RPN10 is not essential, since proteasome-containing vacuolar aggregates were detected at least upon nitrogen starvation treatment in the *RPN10-1* mutant [118]. However, in mammalian cells, the autophagy receptor, p62, is essential for both the formation of proteasome-containing aggresomes and the subsequent autophagy-lysosomal degradation [119,124]. Other proteins, such as the scaffold protein ALFY (autophagy-linked FYVE) and aggrephagy receptor NBR1, were discovered to be present in the protein aggregates thought to have a similar function to p62 for assisting aggregation and mediating autophagy degradation [2]. It is yet an unresolved question why both NBR1 and RPN10, the two hitherto plant autophagy receptors identified, are not essential to the formation of proteasome aggregates [118,126]. In yeast, Hsp42 chaperone proteins were found to be essential for the aggregation of genetically impaired proteasomes into IPODs [125]. Whether Hsp42 or yet unknown chaperone proteins are required for plant proteasome aggregates requires further investigation (Figure 3). 

In addition to the presence of soluble and insoluble substances in cells, proteinaceous and membraneless subcellular structures are now considered as a third biophysical state of proteins assembled through LLPS. LLPS not only develops protein aggregates, but also forms many functional subcellular structures, such as centrosome, the nucleolus, Cajal bodies, and P-bodies [1]. Interestingly, p62 has been recently found to form proteolytically active nuclear condensates in mammalian cells that are generated through LLPS. Within these condensates, ubiquitylated substrates, 26S proteasomes, three enzymes involved in ubiquitylation, and DUBs are assembled along with p62 into a concentrated droplet for the efficient proteolysis of nuclear proteins and unassembled proteasomal subunits [127]. Therefore, active proteasome condensates, PSGs, JUNK-like proteasome aggregates, and proteasome IPODs, may represent LLPS-regulated biophysical dynamicity of proteasomes [114] (Figure 3).

## 3. Proteolytic Control of Autophagy Flux

Proteasome-containing condensates are enriched with ubiquitin and ubiquitin-chains given that many of its subunits are ubiquitylation substrates [118,124,125,129]. Since polyubiquitin chains can be recognized by autophagy receptors, such as p62, NBR1, and RPN10, for proteaphagy degradation. This is evolutionarily harmful to normal cellular growth, under which the UPS controls the most protein turnover events [10]. Therefore, the activity of the autophagy is tightly controlled at multi-levels that include transcriptional, post-transcriptional, and post-translational regulations [130]. Since this review focuses on the reciprocal regulation between the UPS and autophagy-mediated degradations, we have updated below the current research state regarding the proteolytic control of autophagy flux by the UPS.

**Figure 4:**
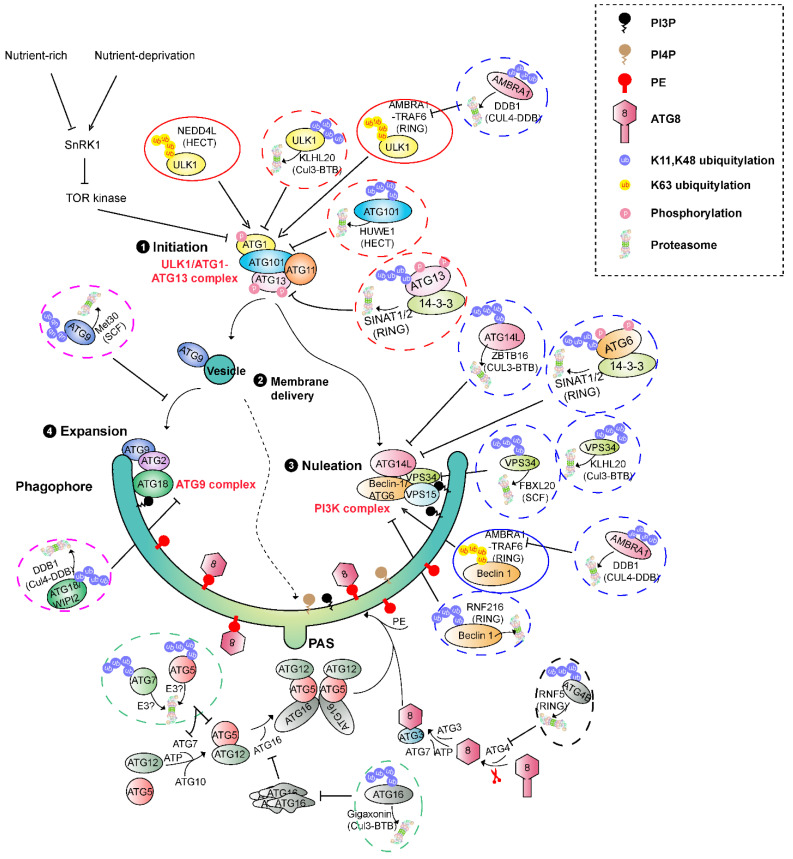
A schematic diagram showing the structural assembly of phagophore and UPS-mediated stability control. Please refer to the text for the detailed process of phagophore initiation, nucleation, and expansion. The UPS-mediated stability control of each component is also described in the text.

In general, autophagy is referred to as macroautophagy that involves the de novo formation of double-membraned vesicles, i.e., autophagosomes, for delivering autophagy cargo into lysosomes in mammalian cells or vacuoles in yeast and plant cells, where the substances are degraded by the resident proteases [19]. Proteaphagy belongs to this type. The formation of autophagosomes undergoes several steps of membrane processing that can be sequentially divided into phagophore initiation, nucleation, elongation, maturation, and fusion with the lysosome or vacuole [19,131] (Figure 4). According to the biochemical functions, six entities involving about 20 ATG proteins can be temporally and spatially separated: (1) ATG1-ATG13-protein kinase complex, (2) ATG9-containing vesicles, (3) ATG9-ATG2-ATG18 transmembrane complex, (4) phosphatidylinositol 3-kinase (PI3K) complex composed of ATG6-ATG14-VPS15-VPS34, and (5–6) two ubiquitin-like conjugation pathways involving one E1 (ATG7), two E2s (ATG3 and ATG10), one E3 (ATG5-ATG12-ATG16), and two ubiquitin-like proteins (ATG8 and ATG12) [18,19]. Interestingly, many members involved in these entities have been discovered as a ubiquitylation substrate for degradation in the 26S proteasome (Figure 4, Table 1), further demonstrating a role of the ubiquitin signal in switching the stress response [82].

### 3.1. Members in Phagophore Initiation

#### 3.1.1. ATG1-ATG13 Kinase Complex

The ATG1-ATG13 kinase complex is the first upstream factor that senses nutritional cues and recruits downstream ATG proteins, such as ATG9, to initiate autophagy [18,19,151]. The plant ATG1-ATG13 complex is formed by ATG1, ATG11, ATG13, and ATG101 [152,153]. The ATG1 orthologue in mammals is termed ULK1 (Unc51-Like Kinase1). The ULK1-ATG13 complex comprises ULK1, ATG13, FIP200 (focal adhesion kinase family interacting protein of 200 kDa), and ATG101 [154,155,156]. Under nutrient-rich conditions, the ATG1-ATG13 complex is dissembled due to ATG13 phosphorylation by target of rapamycin (TOR), a master negative nutrient sensor [157,158]. However, TOR is deactivated by starvation or rapamycin inhibition [159]. TOR inhibition dephosphorylates ATG13, thus promoting and stabilizing the assembly of the ATG1-ATG13 complex, which in turn initiates autophagy [158]. In addition to phosphorylation regulation by TOR, the function of the ATG1-ATG13 kinase complex in both plants and mammals has been discovered under the control of UPS-mediated degradation.

In mammals, at least two components of the ULK1-ATG13 complex have been identified as ubiquitylation substrates. Two ubiquitin-E3 ligases, a mono-subunit RING E3 Tumor necrosis factor Receptor-Associated Factor (TRAF) 6 and a Cul3-BTB E3 Cul3^KLHL20^, target ULK1 for K63 and K48 polyubiquitylation, respectively [132,133]. The former ubiquitylation stabilizes ULK1 and is promoted by AMBRA1 (Autophagy and Beclin1 Regulator 1) [132]. AMBRA1 is a WD40-repeat domain-containing proteins with broader binding partners, including Cul4 and Cul5 E3 ligases. Association with Cul4 promotes the autoubiquitylation-mediated degradation of itself. Upon the initiation of autophagy, the activity of ULK1 activates the dissociation of AMBRA1 from a Cul4 E3 ligase complex [139]. Free AMBRA1 is either recruited by TRAF6 to function as a cofactor for enhancing TRAF6-mediated K63 ubiquitylation of ULK1 [144], or inhibits a Cul5 E3 to stabilize the mTOR inhibitor DEPTOR [139]. This positive feedback mediated by AMBRA1 enhances autophagy induction. 

However, prolonged autophagy activity would lead to unrestrained cellular degradation, including proteaphagy, resulting in detrimental effects on cell survival [160,161]. ULK1 turnover is activated by Cul3^KLHL20^-mediated K48 ubiquitylation upon the extension of the starvation period, which provides a negative feedback loop to control the autophagy activity [133]. ULK1 is also targeted for degradation by a third ubiquitin E3 ligase (HECT type), NEDD4L (neural precursor cell-expressed developmentally down-regulated 4–like), for fine-tuning oscillatory activation of autophagy during prolonged stress [134,135]. Like the turnover of ULK1 in controlling the activity of the ULK1-ATG13, the stability of ATG101 is also controlled by a UPS pathway. A HECT E3 ligase, termed the HECT, UBA, and WWE domain containing E3 ubiquitin protein ligase (HUWE)1, was found to target ATG101 for ubiquitylation and degradation in cancer cells for suppressing autophagy [138].

In plants, two mono-subunit RING E3 ligases, SEVEN IN ABSENTIA of *Arabidopsis thaliana* (SINAT1,2), have been discovered to target the ubiquitylation and degradation of ATG13 under nutrient-rich conditions [136]. Upon the degradation of ATG13, the ATG1-ATG13 complex is destabilized, thus terminating autophagy. On the contrary, SINAT6, a RING-truncated SINAT paralog with no ubiquitin E3 ligase activity, stabilizes the ATG1-ATG13 complex by competing SINAT1/2 for binding with ATG13 through the TRAF domain [136]. Further studies of the same group showed that two additional ATG13-interacting proteins, 14-3-3λ and 14-3-3κ, are involved in the turnover of ATG13 [137]. Under nutrient-rich conditions, phosphorylation of ATG13 by TOR facilitates its association with 14-3-3 proteins, which promotes the recruitment of SINAT1/2 for ubiquitination and degradation [137] (Figure 4). However, starvation-induced deactivation of TOR kinase dephosphorylates and stabilizes ATG13 by blocking its interactions with 14-3-3 and SINAT1/2. Interestingly, two TRAF-domain containing homologous proteins, TRAF1a and b (also known as MUSE14 and MUSE13, respectively), interact with ATG13 and SINAT6 to form a stable TRAF1–SINAT6–ATG13 complex that is promoted by ATG1-mediated phosphorylation [137]. This positive feedback loop accelerates the initiation of autophagy upon nutrient depletion. 

#### 3.1.2. ATG9-Containing Vesicles

ATG9 is the only membrane protein among the ATG proteins. It has six transmembrane domains with its N- and C-terminal ends that are both present in the cytosol [162]. ATG9-containing vesicles provide the source of membranes in initiating and/or growing phagophore through trafficking between a phagophore assembly site (PAS) and peripheral organelle sites, such as the Golgi apparatus, endosomes, and mitochondria [162,163,164,165]. In yeast, the interaction between the scaffolding protein ATG17 (FIP200) and ATG9 tethers ATG9 vesicles where it acts as a central hub for gathering multiple ATG proteins, such as the PI3K complex for forming the PAS [166]. This interaction is inhibited by the two regulatory subunits ATG31 and ATG29 but is activated by the ATG1–ATG13 subcomplex. Although ATG13 does not interact with the conserved region of ATG9, its interaction with ATG17 opens the ATG9-binding site on ATG17 [166]. In mammals, starvation-induced ATG9A vesicles deliver the phosphatidylinositol-4-phosphate (PI4P) kinase, PI4KIIIβ, to an autophagosome initiation site on ER for promoting local PI4P production. PI4KIIIβ and PI4P were proposed to recruit the ULK1-ATG13 complex into the autophagosome initiation site [167]. In plants, the autophagosome initiation site was reported to emerge on ER driven by ATG9 vesicles [168]. However, the detailed mechanism underlying plant autophagosome initiation is still missing [169]. A recent study on Arabidopsis showed that PI4P produced by a plasma membrane-localized PI4Kα1 is involved in the assembly and the elongation of the phagophore. Lacking PI4P suppresses ATG8 lipidation, but not the assembly of early phagophore initiation complexes [170].

Consistent with the role in initiating autophagy, a UPS-mediated degradation of ATG9 by SCF^Met30^ in yeast significantly attenuates the autophagy activity [140]. Interestingly, the recognition of ATG9 by the F-box protein, Met30, explicitly occurs at the peripheral sites rather than at the PAS, probably blocked by other ATG9-interacting partners, such as ATG17 [166]. The ubiquitylation and degradation of ATG9 is only activated under nutrient-rich conditions and is profoundly limited during nitrogen starvation. Therefore, SCF^Met30^-mediated ATG9 ubiquitylation and degradation serves as a switch of autophagy [140].

### 3.2. Members in Phagophore Nucleation and Expansion

#### 3.2.1. PI3K Complex

The PI3K complex is composed of four members, a class III phosphatidylinositol-3-kinase (PI3K) vacuolar protein sorting 34 (VPS34), ATG6/VPS30/Beclin1, ATG14/VPS38, and VPS15 [18,19]. In mammals, its activity is regulated by ULK1 through multiple pathways from recruitment to activation. Activation of VPS34 allows the local production of phosphatidylinositol 3-phosphate (PI3P), which is necessary for nucleation and expansion of autophagosome membranes by recruiting multiple downstream autophagy components [171,172].

Among this complex, VPS34 and ATG14 are targeted by SCF^FBXL20^ and Cul3^ZBTB16^ for ubiquitylation and degradation in response to DNA damage and G protein-coupled receptor signaling, respectively [141,147]. VPS34 was also discovered to be ubiquitylated by Cul3^KLH20^ in HeLa cells [133]. In addition, two E3 ligases, including one HECT Nedd4 and one mono-subunit RING RNF216, recognize Beclin1 for K11 and K48 ubiquitylation and subsequent degradation [142,143]. Interestingly, Cul4-DDB1-AMBRA1 and TRAF6 E3 ligases target Beclin1 for K63-ubiquitylaiton, preventing its degradation in proteasome [144,145]. 

It is not uncommon that one ubiquitin E3 ligase can target multiple substrates. Like TRAF6 and Cul3^KLH20^, the two E3 ligases, SINAT1 and SINAT2, were found to target not only ATG13 but also the plant Beclin 1 orthologue, ATG6, for ubiquitylation and degradation [136,137,146]. It is yet unknown how and whether these E3 ligases ubiquitylate different ATG proteins locally in phagophore or separately in free cytosolic forms before they are recruited into each complex. Both cases could be possible, because SCF^Met30^ is found to target ATG9 ubiquitylation at the peripheral sites while Cul3^KLH20^ seems to work locally in the phagophore site [133,140]. If the latter case happens, it would be intriguing to further investigate whether other members in each complex could also be targeted for ubiquitylation and/or degradation by TRAF6 and SINAT1/2 in mammals and plants, respectively. For example, Cul3^KLH20^ not only directs ULK1 and VPS34, but also directs members in each complex, including ATG13, Beclin1, and ATG14, for ubiquitylation and degradation [133]. Nedd4 was also co-immunoprecipitated with VPS34 by Myc-tagged Beclin1 proteins in HeLa cells [143]. However, Nedd4 has no effect on the stability of VPS34, suggesting a functional specificity of different E3 ligases. However, how this specificity is regulated remains elusive. Furthermore, how the K11 and K48 ubiquitylated proteins are released from the phagophore and delivered into the proteasome for degradation is also unknown.

#### 3.2.2. ATG9-ATG2-ATG18 Complex

The ATG9-ATG2-ATG18 complex is not only important for establishing the phagophore–ER contact site, but also for phagophore expansion through Atg2-Atg18-mediated lipid transfer [173,174]. In mammals, the WIPI2/ATG18B family positively regulates LC3 lipidation [175].

A Cul4^DDB1^ E3 ligase has been found to target WIPI2 (mammal version of ATG18) for ubiquitylation and degradation via its DDB1 (damage-specific DNA binding protein 1) substrate receptor upon mitosis induction. Consequently, the autophagy is suppressed [148].

### 3.3. Members in ATG8/LC3 Lipidation

Two ubiquitylation-like pathways are present in yeast, mammals, and plants, designed for the lipidation of the ubiquitin-like protein, LC3 (mammals) or ATG8 (yeast and plants), onto the phosphatidylethanolamine (PE) of the phagophore. In addition to cargo selection through interacting with autophagy receptors [176], the conjugation of LC3/ATG8 is essential for the expansion, closure, and delivery of autophagosomes [177,178]. At the beginning of these two pathways, the ATG8 precursor needs to be proteolytically processed by the protease ATG4 for exposing its C-terminal end glycine [179]. Both processed ATG8s and another ubiquitin-like protein, ATG12, are activated through their C-terminal end glycine residue by the ATP-dependent E1-like ATG7 enzyme. Activated ATG8 is then conjugated with an E2-like ATG3 to make a heteromeric ATG8-ATG3 intermediated conjugate, while ATG12 is covalently linked onto ATG5 by another E2-like protein, ATG10. Two copies of ATG5-ATG12 conjugate and ATG16 bind together to form a hexameric ligase for targeting ATG8 from its ATG8-ATG3 intermediated conjugate onto PE via an ether linkage (Figure 4).

Up to now, ATG4, ATG7, and ATG16 have been found as a ubiquitylation substrate subject to proteasomal degradation. A membrane-associated mono-subunit RING E3 ligase, RNF5, involved in ER-associated protein degradation (ERAD), was discovered in *Caenorhabditis elegans* and mice. Given the link between autophagy and ER stress, it has been proposed that some of the ubiquitin ligases responsible for the ER stress response could also affect distinct phases of the autophagy process. Interestingly, RNF5 was discovered to target the major mammalian ATG4 isoform, ATG4B, for ubiquitylation and degradation in mice [149]. This process is primarily limited to the membrane domains since RNF5 is membrane-associated. However, the RNF5-mediatied control of ATG4B and the subsequent LC3 processing significantly suppresses the basal level of autophagy under normal growth conditions [149]. On the contrary, a UPS-mediated stability control of ATG16 is essential for LC3 lipidation [150]. Deletion of an ATG16-binding BTB protein, Gigaxonin, induces ATG16L1 aggregation, due to its stabilization, and impairs LC3 lipidation, which results in an altered lysosomal fusion and the elimination of p62 degradation [150]. This is consistent with a previous discovery showing that the overexpression of ATG16L inhibits autophagosome formation. An over-accumulation of ATGL16 probably depletes the hypothetical ATG16L-binding factor that is required for membrane localization of the ATG16L complex, rather than affects the stoichiometry of the hexameric ATG5-ATG12-ATG16 E3 ligase [180]. In addition to ATG4 and ATG16 that are found to be ubiquitylated in mammals, we recently discovered that ATG7 is subject to ubiquitylation-mediated proteasomal degradation, although the identification of its ubiquitin E3 ligase(s) is still awaiting [95].

## 4. Conclusions

With the advance in genomics sequencing technology, it is not a bottleneck any longer to identify the most, if not all, of the protein-coding genes of an organism. However, due to complicated gene expression regulation, various types of post-translational modifications, complex and dynamic interaction networks, and other levels of yet unknown mechanisms, the plasticity of cellular and organismal growth and its development is far more complicated than what we have known. The recently discovered interplay between the two major protein degradation systems, the UPS and the autophagy, may provide an excellent platform to systematically explore how the intracellular proteome is dynamically balanced through various degradation-mediated checkpoint processes. The emerging new biophysical state of proteasomes and the UPS in reaction to condensate droplets provides an intriguing new direction for further understanding of the molecular and biochemical mechanisms underlying the biological function of the UPS and the dynamics of the proteasome. Given its size and abundance, the 26S proteasomes may dynamically shift in its different forms of liquid droplets via LLPS. In the future, it is worth investigating the role of ubiquitin, different types of ubiquitin chains, and numerous proteasome-associated proteins, including chaperons, in this shift. It was discovered that Cdc48/p97, a highly conserved AAA+ ATPase, collaborates with the proteasome by extracting ubiquitylated proteins from macromolecular complexes and membranes with its unfoldase activity [181]. This activity may be active in the proteasomal degradation of proteasome subunits in JUNQ bodies. Thus, the interplay between the UPS and autophagy may also involve Cdc48. There are many unknown regulatory mechanisms present in these two protein degradation systems. One big challenge ahead would be the molecular and biochemical characterization of the large group of ubiquitin E3 ligases, particularly in flowering plants, whose UPS has been dramatically expanded [182,183]. However, with new imagining tools, advances in structural biology, in-depth proteomics, and high-throughput functional genomics studies, more exciting discoveries will be uncovered to shed light on how the life process is maintained via protein death [184]. 

## Figures and Tables

**Table 1:** List of ATG proteins identified as a UPS substrate.

Protein	Biochemical Function	E3 Ligase		Organism	Reference
		Name	Type		
**1. Members in phagophore initiation**					
ULK1	ATG1-ATG13 complex	TRAF6	RING	Human	[132]
ULK1	ATG1-ATG13 complex	Cul3-KLHL20	Cul3-BTB	Human	[133]
ULK1	ATG1-ATG13 complex	NEDD4L	HECT	Human	[134,135]
ATG13	ATG1-ATG13 complex	SINAT1/2	RING	Arabidopsis	[136,137]
ATG101	ATG1-ATG13 complex	HUWE1	HECT	Human	[138]
AMBRA1	Cul5 suppressorTRAF6 activator	Cul4-DDB1-AMBRA1	Cul4-DDB1	Human	[139]
ATG9	Phospholipid scramblase	SCF-Met30	SCF	Yeast	[140]
**2. Members in phagophore nucleation and expansion**					
VPS34	PI3K	SCF-FBXL20	SCF	Human	[141]
VPS34	PI3K	Cul3-KLHL20	Cul3-BTB	Human	[133]
Beclin1/ATG6	PI3K	RNF216	RING	Human	[142]
Beclin1/ATG6	PI3K	Nedd4	HECT	Human	[143]
Beclin1/ATG6	PI3K	Cul4-DDB1-AMBRA1	Cul4-DDB1	Human	[144]
Beclin1/ATG6	PI3K	TRAF6	RING	Human	[145]
Beclin1/ATG6	PI3K	SINAT1/2	RING	Arabidopsis	[146]
ATG14L	PI3K	Cul3-ZBTB16	Cul3-BTB	Human	[147]
WIPI2/ATG18	PI3K	Cul4-DDB1	Cul4-DDB1	Human	[148]
**3. Members in ATG8/LC3 lipidation**					
ATG4B	Protease	RNF5	RING	Human	[149]
ATG16L	E3	Cul3-Gigaxonin	Cul3-BTB	Human	[150]
ATG7	E1	---	---	Arabidopsis	[95]

## Data Availability

Not applicable.

## Abbreviations

ALFY	Autophagy-Linked FYVE
AMBRA1	Autophagy and Beclin1 Regulator 1
ATG	AuTophaGy-Related Proteins
CDC48	Cell Division Control Protein 48
CP	Core Particle
Cryo-EM	Cryo-Electron Microscopy
CSN	COP9 SigNalosome
DDB1	Damage-Specific DNA Binding protein 1
DUB	DeUBiquitinase
eIF3	Eukaryotic Initiation Factor 3
ER	Endoplasmic Reticulum
ERAD	ER-Associated Protein Degradation
FIP200	Focal Adhesion Kinase Family Interacting Protein of 200 kDa
GFP	Green Fluorescent Protein
HSP42	Heat Shock Protein 42
HUWE1	HECT: UBA and WWE Domain Containing E3 Ubiquitin Protein Ligase 1
IPOD	Insoluble Protein Deposit
JUNQ	JUxtaNuclear Quality Control
LC3	Microtubule-Associated Protein 1 Light Chain 3
LLPS	Liquid–Liquid Phase Separation
MG132	Carbobenzoxy-Leu-Leu-leucinal
MTOC	MicroTubule-Organizing Center
NBR1	Next to BRca1
NEDD4L	NEural Precursor Cell-Expressed Developmentally Down-regulated 4–Like
OB	Oligonucleotide/oligosaccharide-Binding
P62/SQSTM1	SeQueSTosoMe 1
PA200	Proteasome Activator 200 kDa
PAS	Phagophore Assembly Site
PC	Proteasome/Cyclosome
PCI	Proteasome–CSN–eIF3
PE	Phosphatidyl Ethanolamine
PI3K	PhosphatidylInositol-3-Kinase
PI3P	PhosphatidylInositol 3-Phosphate
PI4P	PhosphatidylInositol 4-Phosphate
PQC	Protein Quality Control
PSGs	Proteasome Storage Granules
RING	Really Interesting New Gene
RP	Regulatory Particle
RPN	Regulatory Particle Non-ATPase
RPT	Regulatory Particle Triple-A ATPases
SCF	Skp1-Cullin1-F-box
SINAT	Seven in Absentia of Arabidopsis Thaliana
TORC1	Target Of Rapamycin Complex 1
TRAF	Tumor Necrosis Factor Receptor-Associated Factor
ULK1	Unc51-Like Kinase1
Ump1	Ubiquitin-Mediated Proteolysis 1
UPS	The Ubiquitin-26S Proteasome System
VPS34	Vacuolar Protein Sorting 34
